# Spatiotemporal Patterns and Risk Factors for Newcastle Disease Virus among Chickens in a Tanzania Live Bird Market

**DOI:** 10.1155/2024/5597050

**Published:** 2024-03-08

**Authors:** John B. Tsaxra, Rodrigo A. Gallardo, Celia Abolnik, Augustino A. Chengula, Peter L. M. Msoffe, Amandus P. Muhairwa, Thandeka Phiri, James R. Mushi, Nadira Chouicha, Esther L. Mollel, Huaijun Zhou, Terra R. Kelly

**Affiliations:** ^1^Department of Microbiology, Parasitology and Biotechnology, Sokoine University of Agriculture, Morogoro, Tanzania; ^2^Feed the Future Innovation Lab for Genomics to Improve Poultry Project, Davis, USA; ^3^Livestock Training Agency, Mabuki Campus, Mwanza, Tanzania; ^4^School of Veterinary Medicine, University of California, Davis 95616, USA; ^5^Faculty of Veterinary Sciences, University of Pretoria, Pretoria, South Africa; ^6^Department of Veterinary Medicine and Public Health, Sokoine University of Agriculture, Morogoro, Tanzania; ^7^Department of Animal Physiology, Biochemistry, Pharmacology, and Toxicology, Sokoine University of Agriculture, Morogoro, Tanzania; ^8^Department of Animal Science, University of California, Davis 95616, USA; ^9^EpiEcos, Flagstaff, Arizona 86004, USA

## Abstract

Village poultry plays a vital role in providing essential nutrition and income for rural communities in Africa. In this context, poultry are often traded through live bird markets (LBMs), which serve as central trading hubs where producers connect with traders and consumers, facilitating the flow of poultry products along the value chain. While they serve as important trading hubs, these markets create an environment where avian pathogens, like Newcastle disease virus (NDV) and avian influenza virus, can easily emerge and spread. Improving our understanding of the epidemiology of NDV in LBMs is important for assessing disease risks and identifying factors that contribute to its persistence. Local chickens at the Mawenzi LBM in Morogoro municipality were surveyed for NDV presence, its temporal and spatial distribution, and risk factors for NDV infection. Twenty-three percent of 659 local chickens sampled over a 1-year period were positive for NDV based on PCR. Increased odds of NDV infection were identified in chickens that had been in the market for 2 or more days prior to sampling and during the period extending from August through October. Four significant spatiotemporal clusters of NDV-positive chickens encompassing 13 villages were detected between August and October 2020, illustrating geographic hotspots of infection when NDV was most prevalent. Similar to the other LBMs, this market had enclosures with high densities of birds of mixed species, limited biosecurity, and the presence of birds with observable illness. Bird traders who source the chickens from the villages, described long transit times in mixed enclosures with limited sanitation practices and without consideration of sick birds or vaccination status prior to arriving at the LBM. This study highlights the need to invest in improvements to infrastructure and biosecurity for LBMs as well as training opportunities for increasing traders' knowledge on hygiene and sanitation practices, animal welfare, and poultry biosecurity measures.

## 1. Introduction

Traditional poultry keeping is widely practiced in rural areas of low and middle-income countries throughout the world. Local chicken production is a low-input system requiring minimal investment [1] making it accessible to resource-poor communities [2]. Even households without land can raise chickens because the birds can be sustained through scavenging for food on communal village land [3–8]. Village poultry can provide households with earnings and a source of relatively inexpensive protein and nutritionally rich food, thereby contributing to nutritional security and poverty alleviation [6]. Furthermore, chickens play an important role in sociocultural practices as they are offered as gifts for friends and family, as a welcome meal for visitors, and for other rituals [9].

Local poultry production is common among households in Tanzania, especially in resource-limited communities. Smallholder poultry producers face a number of challenges when rearing village poultry flocks, including predation, poor husbandry, as well as disease [5, 10]. Newcastle disease (ND), in particular, is an important constraint to traditional poultry keeping in sub-Saharan Africa with high mortalities among local flocks every year [11–14]. In Tanzania, vaccination against ND is utilized as a means of controlling the disease [11]; however, it is challenging to implement effective vaccination programs in remote village settings.

Newcastle disease is caused by avian *orthoavulavirus* 1 (AOaV-1; formerly avian paramyxovirus-1) which belongs to the genus Avulavirus, subfamily Paramyxovirinae, and family Paramyxoviridae [15]. Newcastle disease virus (NDV) is a negative sense single-stranded RNA-enveloped virus [16]. AOaV-1 viruses consist of virulent and avirulent strains which fall into a single serotype. The phylogenetic analysis of the fusion (F) gene shows that NDV can be grouped into two classes, Class I and Class II, with the former primarily including the avirulent viruses most commonly found in wild birds and the latter encompassing the virulent viruses found in both wild birds and poultry. Class II is further divided into 20 genotypes [17]. Furthermore, NDV can be divided into pathotypes; lentogenic (low virulence), mesogenic (moderate virulence), and velogenic (high virulence) [18]. All pathotypes of NDV occur in rural poultry, but velogenic strains are most commonly reported in Africa [19–21]. Velogenic Newcastle disease virus (vNDV) is considered endemic among village chickens in most low-middle income countries [22, 23], and epidemics of vNDV result in devastating economic losses for the poultry producers [24].

Over the past few decades, investigators in Tanzania have isolated and characterized both lentogenic and velogenic strains of NDVs [20, 21, 25, 26], some of which were detected in live bird markets (LBMs). Previous research has revealed a seroprevalence of 26% for ND in major LBMs in two regions of Tanzania, Morogoro and Dar es Salaam [27]. LBMs congregate live chickens from smallholder farmers located throughout the country. They provide a platform for small-scale poultry producers to access wider markets beyond their immediate communities. Local chickens are sold largely through the LBMs because of the lack of a cold chain for the distribution of chilled fresh meat directly to the markets in urban areas [21]. Because these markets maintain a constant influx of new birds and have minimal biosecurity, they can act as a source of pathogens, such as NDV and influenza A virus, for chickens and other species in the market, and also pose a risk to humans due to zoonotic viruses affecting livestock [21, 28–31]. In these settings, indigenous poultry can become infected and serve as reservoirs for more susceptible exotic breeds in commercial farms [32] as well as wild birds coming into contact with infectious birds and/or materials at the market. Epidemiological studies are therefore increasingly valuable to gain additional insights into the diversity of circulating NDV strains and the ecology of the virus in the market and village settings. This study aimed to investigate the prevalence, spatiotemporal patterns, and risk factors for NDV among chickens in a LBM in Tanzania.

## 2. Materials and Methods

### 2.1. Study Area and Design

Samples were collected weekly over a 1-year period (June 2020–May 2021) from the Mawenzi LBM located in Morogoro municipality in Tanzania, 196 km west of Dar es Salaam, the country's largest city and commercial center, and 260 km east of Dodoma, Tanzania's capital.

The Mawenzi LBM is located within the general food market. The live poultry are sheltered in wood and wire mesh cages stacked on top of each other. Birds are provided with food (maize bran) and water in the cages. There is a small bird slaughtering and processing area within the market that is located next to the bird enclosures that is devoid of water supply and sanitation facilities [21].

As part of this study, the Mawenzi LBM was characterized weekly during each sampling event by collecting data on the number of vendors selling birds, the numbers and types of birds present, and the condition of the birds for sale.

### 2.2. Sample Collection and Processing

Over the period of 1 year, a total of 659 chickens were sampled at the Mawenzi LBM. Oro-cloacal swabs (*n* = 659) and sera samples (*n* = 657) were collected from the chickens. The chickens were randomly selected (first and every sixth count afterward) for sampling each week. Most samples were collected immediately after chickens were offloaded at the market from the villages, but some chickens were sampled after the bird had been in the market for 2 or more days.

The oro-cloacal samples were collected using sterile polyester-tipped swabs (Puritan, USA). The swabs from each bird were placed in individual vials with phosphate buffered saline (PBS) and immediately transported to the laboratory in a cooler to be stored at −80°C until further analysis. Whole blood (approximately 1 mL) was collected from the brachial vein into a 3 mL syringe (23G needle) and the blood was left to clot overnight. Sera were decanted and stored in 1.5 mL Eppendorf tubes at −20°C until hemagglutination inhibition (HI) assays were performed.

During each weekly sampling event, the following data were recorded for each sample: date of sample collection, the sex, health status, source (geographic location), and type of chicken (indigenous or exotic breed) sampled. Data were also collected on the timing of sampling of the chicken (i.e., whether the bird was sampled immediately after it was offloaded at the market for sale or after it had been in the market for 2 or more days).

### 2.3. Questionnaire

Questionnaires were administered to 23 chicken traders (16 middlemen and seven sellers) who consented to participate in the study. The aims of the study were communicated in Swahili, and written informed consent was obtained from all study participants. Questionnaires were administered to collect demographic information on the trader as well as data on the source of the bird (village), whether the chickens were housed together with other bird species at the source and during transit, the traders' criteria for selecting birds to sell in the market, whether chickens were obtained from one source all year-round, length of time spent in transit to the market, length of time spent at the market prior to sale, temporal patterns of illness in the traded birds, and biosecurity practices used by the trader in transporting and housing the birds prior to sale (e.g., cage disinfection).

### 2.4. Serological Analysis

Sera collected from local chickens at the Mawenzi LBM were analyzed for antibodies against NDV using the haemagglutination-inhibition (HI) assay as previously described [33, 34]. The assay was performed according to the recommended standard procedure [35]. In brief, 25 *μ*L of PBS was dispensed into each well of a plastic V-bottomed microtiter plate. A total of 25 *μ*L of serum was placed into the first well of the plate. Twofold dilutions of the 25 *μ*L serum were then made across the plate. Twenty-five microliter (25 *μ*L) of four hemagglutinating units of ND viral antigen (LaSota) was added to each well and the plate was left for 30 min at room temperature. Chicken red blood cells (RBCs) (25 *μ*L of 0.5% (*v*/*v*)) were then added to each well and, after gentle mixing by tapping the sides of the plate, RBCs were allowed to settle at room temperature for 40 min. The HI titer of each serum sample was expressed as the reciprocal of the serum dilution and expressed as the logarithm to base 2 (log_2_). Haemagglutination was assessed by tilting the plates with those wells in which the streaming of RBCs was observed at the same rate as the control wells (positive serum, virus/antigen, and PBS controls) considered positive (inhibition of hemagglutination) [35]. The positive serum and virus/antigen used in the assay were provided by the Virology Laboratory at the Department of Veterinary Microbiology, Parasitology, and Biotechnology of Sokoine University of Agriculture.

### 2.5. Real-Time Reverse Transcription PCR (rRT-PCR)

Total nucleic acid was extracted from oro-cloacal swab samples using the IndiMag Pathogen Kit (Indical Bioscience, USA) in an IndiMag™ 48 instrument, according to the manufacturer's instructions. rRT-PCR was performed with VetMAX Plus RT-PCR kits (Thermo Fisher Scientific, MA, USA) (half-reactions) according to the manufacturer's recommended protocol and a 53°C annealing temperature, with MGB/Fam-labeled NDV L-gene specific primers and probes described by Fuller et al. [36], in a StepOne Plus thermal cycler (Applied Biosystems). Cycle threshold (Ct) values <40 were considered positive for NDV.

### 2.6. Statistical Analyses

The NDV prevalence and seroprevalence and respective 95% confidence intervals were estimated. Latitude and longitude coordinates were assigned to each chicken source based on the center point of the village where the chicken was collected. Temporal and spatial clustering of NDV was evaluated among chickens using Bernoulli model elliptical scanning windows in SaTScan v.10.0 [37–39]. A maximum spatial and temporal cluster size of 25% of the population at risk was used for the spatiotemporal analysis, and overlapping clusters were not permitted. For the analysis, 1 month was set as the time aggregation unit. Clusters were mapped using qGIS [40].

Unadjusted bivariate associations between NDV infection, host factors, and spatiotemporal variables were evaluated using *χ^2^* tests of independence. These variables were further evaluated for multivariable associations with NDV infection using mixed effects logistic regression models to evaluate the influence of putative risk factors on the odds of infection in chickens at the LBM. Factors evaluated for their association with NDV infection (based on PCR results) included sex of the chicken, month of sample collection, geographical (region) origin of the bird prior to being offloaded at the market, and status of chicken at the time of sample collection (new arrival to the market or chicken present for >2 days in the market prior to sample collection). For all multivariable models, putative risk factors with *P*  < 0.20 in the bivariate analyses were evaluated in the mixed effects logistic regression models using the lme4 package in R [41] and retained in the models if *P*  < 0.05. The ID of the chicken trader was included in the model as the cluster variable to account for unmeasured correlation among multiple chickens from one source.

In all models, NDV infection by month was evaluated for a difference in magnitude across months, and significance of effect between months using the likelihood ratio statistic and similar months were collapsed into the following time periods: January–March, April–July, and August–October. Possible confounding variables and interaction effects were evaluated in the model building process. Confounding was evaluated in the model through assessing whether there was a greater than 10% change between unadjusted and adjusted odds ratio estimates for the other variables in the model. Final parsimonious models were selected using Akaike's Information Criterion (AIC) for comparisons between nested and nonnested models. Overall model fit was evaluated using Hosmer–Lemeshow goodness-of-fit test and measures of information criteria. Odds ratios were estimated with 95% confidence intervals. All analyses were performed using R statistical software version 4.2.2 (October 31, 2022) [42].

## 3. Results

A total of 659 chickens were sampled during a 1-year period (from June 2020 to May 2021) at the Mawenzi LBM in Morogoro municipality. On average, there were 316 birds housed at the market at a given sampling point. The birds were of mixed species, including guinea fowl, ducks, and indigenous chickens. The birds were sourced from villages within the Morogoro, Dodoma, Tabora, Shinyanga, and Manyara regions. On average, one chicken exhibited clinical signs of illness during each weekly sampling event, which ranged from drowsiness, mucoid, and watery discharge from the mouth and nostrils, and diarrhea to swollen eyes, cyanotic comb and wattles, gasping, rales, and torticollis.

The overall NDV seroprevalence and prevalence, based on HI and PCR, among the chickens at the market over the study period were 22.1% (95% CI: 19.2%–25.5%) and 23.8% (95% CI: 21.1%–27.2%), respectively (Table 1). The Ct values for PCR positive samples ranged from 17.6 to 39.3 with a median of 31.1. NDV strains identified through genotyping are reported in a follow-up study [43].

In the multivariable analysis, chickens that had been held at the market for 2 or more days prior to sampling had a three times higher odds of testing positive for NDV as compared to chickens that had just been offloaded for sale at the Mawenzi market (Table 2, OR = 3.0 (95% CI: 1.4–6.8), *P* = 0.007). In addition, the odds of NDV infection were highest during the period extending from August to October followed by the period of April to July relative to the months of November through March (Table 2). Sex of the chicken was not significantly associated with NDV infection among birds in this study.

**Figure 1:**
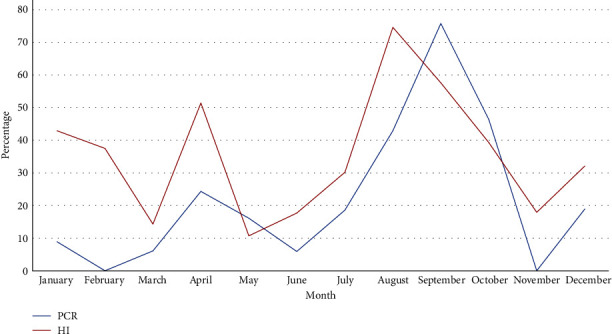
Monthly seroprevalence (by HI) and prevalence (by PCR) of Newcastle disease virus in chickens at the Mawenzi LBM in Morogoro, Tanzania from June 2020 to May 2021.

In evaluating the temporal distribution of NDV infection in the LBM chickens, results revealed a peak in the prevalence of the virus among chickens between August and October (Figure 1).

**Figure 2:**
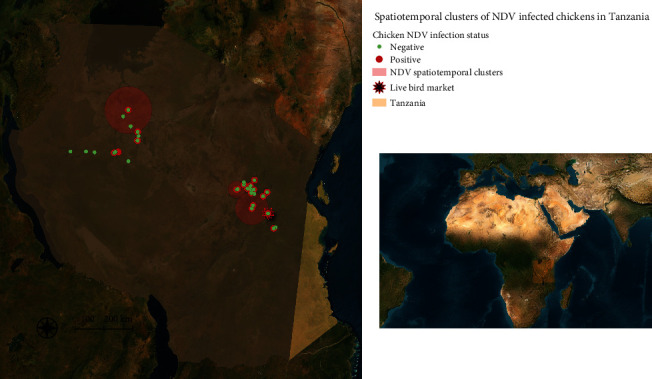
Map showing significant spatiotemporal clusters of Newcastle disease virus infected chickens transported and offloaded at the Mawenzi LBM in Morogoro, Tanzania from June 2020 to May 2021.

Four significant spatiotemporal clusters of NDV-positive chickens (Figure 2) were detected with radii of 0, 83.7, 58.42, and 30.47 km with *P*  < 0.0001, *P*  < 0.0001, *P* = 0.002, and *P* = 0.01, respectively. The clusters, encompassing 13 villages in Morogoro (6), Manyara (1), Dodoma (2), Tabora (3), and Shinyanga (1) regions were detected between August and October 2020.

A total of 23 chicken traders (16 middlemen and seven sellers, all male) in the Mawenzi LBM provided responses to the questionnaire. The data stemming from questionnaires administered to these individuals revealed that chickens from different sources were mixed (pooled) in enclosures before and during transportation to the market. The average transit time of birds from the source to the market was 1–5 days. Middlemen collected and transported birds by public buses and/or trucks within cages with some birds tethered to the outside of the cages when space was limited. Chickens from different sources were mixed during transportation and at the point of sale in the market, including mixing of newly acquired birds with birds that were already present in the market. The questionnaire also revealed that chicken cages (collection and/or transport cages) were not disinfected between bird collections. Chickens were also pooled together at the market regardless of their health status. All of the respondents indicated that they were able to distinguish between ill and healthy birds. The respondents reported that they often encounter ill birds during the collection of chickens. When asked how sick birds are managed, 6% of traders indicated that sick birds were provided herbal remedies at the households. In addition, 18% of traders indicated that ill birds were slaughtered for home use and 76% reported that sick chickens were transported to the market for sale. Regarding the criteria of selection of chickens by the live bird traders, only 12% of the traders considered an ill bird to be unfit for transport and sale at the market. In addition, the traders indicated that vaccination history for the birds was unknown. When asked about seasonality of illness among collected birds, live bird traders reported that illness was most prevalent from June to October, compared to the other periods of the year.

## 4. Discussion

This study revealed that the local chicken trade associated with the Mawenzi LBM in Morogoro Municipality serves as a source of circulating NDV and provides a potential environment for virus transmission and regional spread among chickens and possibly other avian species in Tanzania. The NDV prevalence and seroprevalence among chickens sampled at the Mawenzi LBM for this study were in the range of previously reported estimates in Tanzania. Other ND studies conducted in villages in Tanzania reported seroprevalences of 46.8% [44] and 13.3% [45]. The study conducted by Msoffe et al. [21] revealed a 32% prevalence of NDV in chickens at a LBM. A separate study reported a NDV seroprevalence of 26% in the villages in Njombe and Bahi districts in Tanzania [46]. Chickens at the Mawenzi LBM were sourced from parts of Morogoro, Shinyanga, Tabora, Dodoma, and Manyara regions. In Tanzania, the local poultry trade is unregulated with limited to no biosecurity along the market chain [47]. The absence of active NDV surveillance and biosecurity practices in the trade of local chickens poses a significant challenge for disease prevention and control strategies and may contribute to regular outbreaks of ND in the country [21, 48]. Many studies have illustrated the potential role of unregulated poultry trade through LBMs on ND activity. For example, congregating poultry originating from different areas along with transportation and poor biosecurity measures have been associated with NDV outbreaks in Ethiopia and Kenya [28, 29].

Our findings revealed that, with the exception of 1 month (February), NDV is circulating year-round among the local chickens coming into the LBM providing further evidence of the endemicity of this virus and the role that the LBM plays as a continued source of infection among birds originating from different parts of the country. The temporal and spatiotemporal patterns indicated seasonality of NDV activity with the highest prevalence of NDV infected birds during the period from August to October. This was corroborated by the live bird traders who indicated that illness among the chickens was relatively more frequent during the period from August to October. Unfortunately, we were not able to differentiate whether antibodies in the birds were due to previous exposure or ND vaccination. However, the seasonal pattern of antibody response in the birds was similar to the seasonality of NDV shedding (as measured by PCR) suggesting that the temporal pattern of seropositivity observed in this study may have been due in part to immunity post-infection. Seasonality of NDV activity, as corroborated by other studies [49–54], might be due to the dry weather during the period of August–October in most parts of the country during which poultry food sources are scarce, a stress that can lead to immunosuppression in the chickens. Other studies conducted in East Africa and in Asia have reported similar patterns with ND peaks during and at the end of the dry seasons [55–59]. During this study, there were multiple geographic hotspots of NDV infection implying that there were multiple sources of virus coming into the Mawenzi LBM. Unfortunately, the exact focal point of infection could not be established in this study because chickens were not sampled at homes or farms of origin but rather at the destination market [60]. In this study, the average time spent during transport from the geographic origins of the chickens to Mawenzi LBM was between 1–5 days. This transit time aligns with the length of the incubation period for ND. As a result, susceptible chickens could become infected while in mixed cages during transportation and present to the market with subclinical and/or clinical disease depending on the individual immunity and time of arrival at the market. Mixing of birds during transportation and upon arrival at the LBM has been previously associated with disease spread and outbreaks [28, 31]. The traders' practices of mixing birds from different places and housing them in mixed species cages at the market regardless of their health status may result in contact between infected and susceptible birds, increasing the risk of transmission. In addition, results from this study indicate a higher odds of infection in birds that have been present at the market for 2 or more days highlighting that cohousing of new arrivals with resident market birds is a significant risk factor for NDV spread. The vaccination history of the chickens arriving at the market was unknown further increasing the risk of NDV emergence and spread. Furthermore, the cages used to bring chickens to the LBM were taken back to the villages without disinfection in between bird collections. This heightens the risk of virus carriage back to the villages or from one village to the other via the chicken collection cages. In last, traders reported that sick chickens are often included in those birds selected to be sold in the market, further escalating the risk for disease spread among chickens congregating in the LBMs.

The Mawenzi LBM encompasses a chicken slaughtering service that lacks equipment for proper disposal of the visceral contents and feathers which may perpetuate the dissemination of NDVs and the other pathogens [61] in the market and to the surrounding areas. Not unexpectedly, many studies have found that improper disposal of waste (e.g., carcasses, fecal matter) in market settings is associated with greater incidence of disease among flocks [28, 29, 51]. Many LBMs are open air and do not have the appropriate infrastructure and equipment for sanitary slaughter of birds, and therefore pose a risk for contamination of the premises and spillover of NDVs between market birds and wild birds [21, 62].

The findings from this study are applicable to the other pathogens of importance in poultry in LBM settings. For example, influenza A virus has been found to persist in LBMs as a result of poor or inadequate sanitation, the constant presence of chickens in the market, and the practice of mixing birds in enclosures [21, 63–65]. An increased focus on disease prevention and control measures is needed to mitigate these risks, especially given the impacts of the current unprecedented global high pathogenic avian influenza outbreak among poultry and wild birds.

## 5. Conclusion

This study provides further evidence that ND is endemic in Tanzania and circulates among village chickens year-round with peaks of NDV activity during the dry season. Determining the actual burden of the disease based on the source (focal point) might have been overestimated as chickens from different sources were mixed prior to reaching the market. In this case, point-source sampling is suggested in order to more accurately pinpoint spatial patterns. This study identified several risk factors for NDV among birds entering a LBM that highlight potential points of intervention to mitigate risk. Further outreach to smallholder producers on the importance of ND vaccination is recommended to prevent spread through trade. There is a need for greater focus among the stakeholders (live bird sellers and middlemen) in the poultry value chain regarding best practices for biosecurity during transportation and at the point of sale in the markets to reduce risk of NDV and other high consequence poultry diseases. There is also a need for greater investment in market facilities and equipment to improve biosecurity. In last, policies to ensure that markets are regularly inspected by authorities and meet basic sanitation and safety standards are sorely needed to mitigate risk.

## Figures and Tables

**Table 1:** Summary of seropositive (by HI) and PCR positive samples for NDV, by time at which bird was sampled.

Sample collection time	Total sampled (*n* = 659)	Seropositive samples (*n* = 146)		PCR positive samples (*n* = 157)	
		Ill (%)	Healthy (%)	Ill (%)	Healthy (%)
Immediately after offload	515	3 (2.1)	106 (72.6)	8 (5.1)	95 (60.5)
At 2–5 days after offload	144	12 (8.2)	25 (17.1)	31 (19.7)	23 (14.6)
Overall	659	15 (10.3)	131 (89.7)	39 (24.8)	118 (75.2)

**Table 2:** Host and seasonal factors associated with NDV infection in the chickens sampled at the Mawenzi LBM from June 2020 to May 2021.

Factor	Odds ratio	95% CI	*P*-value
Status of chicken (reference group = chicken as new arrival at market)			
Chicken in market ≥2 days	3.0	(1.4–6.8)	0.007
Time period (reference group = November–March)			
April–July	3.8	(1.3–6.7)	0.014
August–October	25.5	(9.7–66.7)	<0.001

## Data Availability

All data generated in this study are available upon request and uploaded in the USAID Development Data Library.

## References

[1] Branckaert R. D. S. (2007). Avian influenza: the new challenge for family poultry. *World’s Poultry Science Journal*.

[2] Wong J. T., de Bruyn J., Bagnol B. (2017). Small-scale poultry and food security in resource-poor settings: a review. *Global Food Security*.

[3] Sonaiya E. B. (1990). The context and prospects for development of smallholder rural poultry production in Africa. *Proceedings International Seminar on Smallholder Rural Poultry Production*.

[4] Barua A., Yoshimura Y. (1997). Rural poultry keeping in Bangladesh. *World’s Poultry Science Journal*.

[5] Kitalyi A. J. (1998). *Village Chicken Production Systems in Rural Africa: Household Food Security and Gender Issues*.

[6] Guèye E. F. (2000). The role of family poultry in poverty alleviation, food security and the promotion of gender equality in rural Africa. *Outlook on Agriculture*.

[7] Branckaert R. D. S., Gaviria L., Jallade J., Seiders R. W. Transfer of technology in poultry production for developing countries.

[8] Permin A., Peterson G., Riise J. C. (2001). Poultry as a tool for poverty alleviation: in: opportunities and problems related to poultry production at the village level.

[9] FAO (2006). *Première évaluation de la Structure et de l’importance du Secteur Avicole Commercial et Familial au Mali*.

[10] Mwalusanya N. A., Katule A. M., Mutayoba S. K., Mtambo M. M. A., Olsen J. E., Minga U. M. (2002). Productivity of local chickens under village management conditions. *Tropical Animal Health and Production*.

[11] Alders R. G., Bagnol B., Young M. P. (2010). Technically sound and sustainable Newcastle disease control in village chickens: lessons learnt over fifteen years. *World’s Poultry Science Journal*.

[12] Aboe P. A. T., Boa-Amponsem K., Okantah S. A., Butler E. A., Dorward P. T., Bryant M. J. (2006). Free range village chickens on the Accra Plains, Ghana: their husbandry and productivity. *Tropical Animal Health and Production*.

[13] Guèye E. F. (1999). Ethnoveterinary medicine against poultry diseases in African villages. *World’s Poultry Science Journal*.

[14] Sylla M., Traoré B., Sidibé S. (2003). Epidémiologie de la maladie de Newcastle en milieu rural au Mali. *Revue D’élevage et de Médecine Vétérinaire des Pays Tropicaux*.

[15] Kuhn J. H., Wolf Y. I., Krupovic M. (2019). Classify viruses—the gain is worth the pain. *Nature*.

[16] Miller P. J. K., Koch G., Swayne D. E., Swayne D. E., Glisson J. R., McDougald L. R., Nolan L. K., Suarez D. L., Nair V. (2013). Newcastle disease. *Diseases of Poultry*.

[17] Dimitrov K. M., Abolnik C., Afonso C. L. (2019). Updated unified phylogenetic classification system and revised nomenclature for Newcastle disease virus. *Infection, Genetics and Evolution*.

[18] Suarez D. L., Miller P. J., Koch G., Mundt E., Rautenschlein S., Swayne D. E., Boulianne M., Logue C. M. (2020). Newcastle disease, other avian paramyxoviruses, and avian metapneumovirus infections. *Diseases of Poultry*.

[19] Awan M. A., Otte M. J., James A. D. (1994). The epidemiology of Newcastle disease in rural poultry: a review. *Avian Pathology*.

[20] Yongolo M. G., Christensen H., Handberg K., Minga U., Olsen J. E. (2011). On the origin and diversity of Newcastle disease virus in Tanzania. *Onderstepoort Journal of Veterinary Research*.

[21] Msoffe P. L. M., Chiwanga G. H., Cardona C. J., Miller P. J., Suarez D. L. (2019). Isolation and characterization of Newcastle disease virus from live bird markets in Tanzania. *Avian Diseases*.

[22] Spradbrow P. B. (1990). Village poultry and preventive veterinary medicine. *Preventive Veterinary Medicine*.

[23] Spradbrow P. B. (1993). Newcastle disease in village chickens. *Poultry Science Reviews*.

[24] Martin P. A. J., Spradbrow P. B (1992). The epidemiology of Newcastle disease in village chickens. in: Newcastle disease in village chickens, control with thermostable oral vaccines. *International Workshop held in Kuala Lumpur*.

[25] da Silva A. P., Aston E. J., Chiwanga G. H. (2020). Molecular characterization of Newcastle disease viruses isolated from chickens in Tanzania and Ghana. *Viruses*.

[26] Loretu K., Mkaria J. A. (1981). Preliminary report on Newcastle disease pathotypes in Tanzania. *Tanzania Veterinary Bulletin*.

[27] Chiwanga G. H. (2012). *Seroprevalence of selected viral diseases of domestic chickens in Dar es Salaam and Morogoro live bird markets*.

[28] Ipara B. O., Otieno D. O., Nyikal R. A., Makokha S. N. (2019). The role of unregulated chicken marketing practices on the frequency of Newcastle disease outbreaks in Kenya. *Poultry Science*.

[29] Mulisa D. D., W/Kiros M. K., Alemu R. B. (2014). Characterization of Newcastle disease virus and poultry-handling practices in live poultry markets, Ethiopia. *SpringerPlus*.

[30] Munyua P. M., Githinji J. W., Waiboci L. W. (2013). Detection of influenza A virus in live bird markets in Kenya, 2009–2011. *Influenza and Other Respiratory Viruses*.

[31] Ogali I. N., Mungube E. O., Lichoti J. K., Ogugo M. W., Ommeh S. C. (2018). A study of Newcastle disease virus in poultry from live bird markets and backyard flocks in Kenya. *Journal of Veterinary Medicine and Animal Health*.

[32] Olabode A. O., Lamorde A. G., Shidali N. N., Chukwuedo A. A. (1992). Village chickens and Newcastle disease in Nigeria. *Australian Centre for International Agricultural Research Proceedings*.

[33] Allan W., Gough R. (1974). A standard haemagglutination inhibition test for Newcastle disease. (2) Vaccination and challenge. *Veterinary Record*.

[34] Hossain K. M. M., Ali M. Y., Yamato I. (2010). Antibody levels against Newcastle disease virus in chickens in Rajshahi and surrounding districts of Bangladesh. *International Journal of Biology*.

[35] World Organization of Animal Health (WOAH) (2018). Manual of diagnostic tests and vaccines for terrestrial animals. https://www.woah.org/fileadmin/Home/eng/Health_standards/tahm/A_summry.htm.

[36] Fuller C. M., Brodd L., Irvine R. M., Alexander D. J., Aldous E. W. (2010). Development of an L gene real-time reverse-transcription PCR assay for the detection of avian paramyxovirus type 1 RNA in clinical samples. *Archives of Virology*.

[37] Kulldorff M., Athas W. F., Feurer E. J., Miller B. A., Key C. R. (1998). Evaluating cluster alarms: a space–time scan statistic and brain cancer in Los Alamos, New Mexico. *American Journal of Public Health*.

[38] Kulldorff M., Nagarwalla N. (1995). Spatial disease clusters: detection and inference. *Statistics in Medicine*.

[39] Kulldorff M., Huang L., Pickle L., Duczmal L. (2006). An elliptic spatial scan statistic. *Statistics in Medicine*.

[40] QGIS.org (1991). *Quantum Geographic Information System*.

[41] Bates D., Mächler M., Bolker B., Walker S. (2015). Fitting linear mixed-effects models using lme4. *Journal of Statistical Software*.

[42] R Core Team (2022). R: A language and environment for statistical computing.

[43] Tsaxra J. B., Abolnik C., Kelly T. R. (2023). Molecular characterization of Newcastle disease virus obtained from Mawenzi live bird market in Morogoro, Tanzania in 2020–2021. *Brazilian Journal of Microbiology*.

[44] Yongolo M. G. S. (1996). *Epidemiology of Newcastle disease in village chickens in Tanzania*.

[45] Minga U. M., Katule A., Maeda T., Musasa J. Potential and problems of the traditional chicken industry in Tanzania.

[46] Mngumi E. B., Bunuma E. (2022). Seroprevalence and risk factors of Newcastle disease virus in local chickens in Njombe and Bahi districts in Tanzania. *Tropical Animal Health and Production*.

[47] Ringo E. J., Lekule F. P. (2020). Market trends and consumer behaviors and preferences in the Tanzania poultry subsector.

[48] Sultan S., Eldamarany N. M. I., Abdelazeem M. W., Fahmy H. A. (2022). Active surveillance and genetic characterization of prevalent velogenic Newcastle disease and highly pathogenic avian influenza H5N8 viruses among migratory wild birds in Southern Egypt during 2015–2018. *Food and Environmental Virology*.

[49] Spradbrow P. B., Alders R. G., Spradbrow P. B. (2000). The epidemiology of Newcastle disease in village chickens. *SADC Planning Workshop on Newcastle Disease Control in Village Chickens*.

[50] Yongolo M. G. S., Machangu A. M., Minga U. M. (2002). Newcastle disease and Infectious bursal disease among free range village chickens in Tanzania. *Characteristics and Parameters of Family Poultry Production in Africa*.

[51] Njagi L. W., Nyaga P. N., Bebora L. C. (2010). Prevalence of Newcastle disease virus in village indigenous chickens in varied agro-ecological zones in Kenya. *Livestock Research for Rural Development*.

[52] Zeleke A., Sori T., Gelaye E., Ayelet G. (2005). Newcastle disease in village chickens in Southern and Rift Valley districts in Ethiopia. *International Journal of Poultry Science*.

[53] Otim M. O., Christensen H., Jørgensen P. H., Handberg K. J., Bisgaard M. (2004). Molecular characterisation and phylogenetic study of Newcastle disease virus isolates from recent outbreaks in eastern Uganda. *Journal of Clinical Microbiology*.

[54] Kemboi D. C., Chegeh H. W., Bebora L. C. (2013). Seasonal Newcastle disease antibody titer dynamics in village chickens of Mbeere dIstrict, Eastern Province, Kenya. *Livestock Research for Rural Development*.

[55] Geresu M. A., Elemo K. K., Kassa G. M. (2016). Newcastle disease: seroprevalence and associated risk factors in backyard and small-scale chicken producer farms in Agarfa and Sinana districts of Bale zone, Ethiopia. *Journal of Veterinary Medicine and Animal Health*.

[56] Chaka H., Goutard F., Bisschop S. P. R., Thompson P. N. (2012). Seroprevalence of Newcastle disease and other infectious diseases in backyard chickens at markets in eastern shewa zone, Ethiopia. *Poultry Science*.

[57] Asadullah M., Spradbrow P. B. (1992). Village chickens and Newcastle disease in Bangladesh. *Newcastle Disease in Village Chickens, Control with Thermostable Oral Vaccines*.

[58] George M. M. (1991). Epidemiology of Newcastle disease in rural Uganda.

[59] Mishra U., Spradbrow P. B. (1992). Present status of poultry in Nepal. *Newcastle Disease in Village Chickens, Control with Thermostable Oral Vaccines*.

[60] Musa U., Abdu P. A., Dafwang I. I. (2009). Seroprevalence, seasonal occurrence and clinical manifestation of Newcastle disease in rural household chickens in Plateau state, Nigeria. *International Journal of Poultry Science*.

[61] Kariithi H. M., Ferreira H. L., Welch C. N. (2021). Surveillance and genetic characterization of virulent Newcastle disease virus subgenotype V.3 in indigenous chickens from backyard poultry farms and live bird markets in Kenya. *Viruses*.

[62] World Bank (2009). Global study of livestock markets, slaughterhouses and related waste management systems.

[63] Fournié G., Guitian F. J., Mangtani P., Ghani A. C. (2011). Impact of the implementation of rest days in live bird markets on the dynamics of H5N1 highly pathogenic avian influenza. *Journal of the Royal Society Interface*.

[64] Leung Y. H. C., Lau E. H. Y., Zhang L. J., Guan Y., Cowling B. J., Peiris J. S. M. (2012). Avian influenza and ban on overnight poultry storage in live poultry markets, Hong Kong. *Emerging Infectious Disease*.

[65] Martin V., Zhou X., Marshall E. (2011). Risk-based surveillance for avian influenza control along poultry market chains in South China: the value of social network analysis. *Preventive Veterinary Medicine*.

